# Blur Unblurred—A Mini Tutorial

**DOI:** 10.1177/2041669518765850

**Published:** 2018-04-18

**Authors:** Hans Strasburger, Michael Bach, Sven P. Heinrich

**Affiliations:** Institute of Medical Psychology, Ludwig-Maximilians-Universität, München, Germany; Department of Medical Psychology and Medical Sociology, Georg-August University, Göttingen, Germany; Section for Functional Vision Research, Eye Center at Medical Center, University of Freiburg, Freiburg im Breisgau, Germany; Faculty of Medicine, University of Freiburg, Freiburg im Breisgau, Germany

**Keywords:** blur, acuity, accommodation, spurious resolution, diopters, optometry, optics

## Abstract

Optical blur from defocus is quite frequently considered as equivalent to low-pass filtering. Yet that belief, although not entirely wrong, is inaccurate. Here, we wish to disentangle the concepts of *dioptric* blur, caused by myopia or mis-accommodation, from blur due to low-pass filtering when convolving with a Gaussian kernel. Perhaps surprisingly—if well known in optometry—the representation of a blur kernel (or point-spread function) for dioptric blur is, to a good approximation and disregarding diffraction, simply a cylinder. Its projection onto the retina is classically referred to as a *blur circle*, the diameter of which can easily be deduced from a light-ray model. We further give the derivation of the relationship between the blur-disk’s diameter and the extent of blur in diopters, as well as the diameter’s relation to the near or far point, and finally its relationship to visual acuity.

## Introduction

A while ago we were wondering whether the low signal amplitudes in fMRI retinotopic mapping and visual evoked potential (VEP) recording from a psychiatric patient could be due simply to a severe lack of correct optical accommodation. Blur reduces retinal contrast (at higher spatial frequencies), which, in turn, should decrease evoked signal amplitude (in VEPs: Strasburger, Murray, & Remky, 1993; Strasburger, Scheidler, & Rentschler, 1988; in fMRI: Boynton, Demb, Glover, & Heeger, 1999). Yet a direct test would be more meaningful. And since inserting plus lenses in our fMRI stimulus goggles would also blur any fixation mark and thus impede fixation, the question arose what the appropriate kernel for digital blurring of the stimulus images would be.

Optical blur from defocus is often considered as equivalent to low-pass filtering (e.g., McAnany, Alexander, Lim, & Shahidi, 2011; Meinecke & Kehrer, 2007; Petrova, Žanete, Cikmačs, & Kassaliete, 2017; Wood, 1983). Yet that belief, although not entirely wrong, is inaccurate. So, here in this short, tutorial-like note we wish to disentangle *dioptric* blur, caused by myopia or mis-accommodation, from blur by low-pass filtering including convolution with a Gaussian kernel. Perhaps surprisingly—if well known in optometry—the blur kernel (or point-spread function [PSF]) for dioptric blur is (to a good approximation and disregarding diffraction) simply a cylindrical disk. Its projection onto the retina is classically referred to as a *blur circle* (see Figure 2(a) which is discussed later), the diameter of which can be easily deduced from a light-ray model. The derivation is given later, where we further derive the relationship between the blur-disk’s diameter and the extent of blur in diopters, and the diameter’s relation to the near or the far point. Finally, we explore the relationship of defocus to visual acuity based on an empirical approach by Blendowske (2015). Note from the outset that we only consider defocus here, not blur in general. The actual PSF is a superposition of the PSF from defocus with the PSFs from diffraction, astigmatism, higher order aberrations—which all depend on pupil diameter—and the idealized, needle-like PSF. It looks, in particular at low levels of mis-accommodation, very different from a cylinder. Examples, for 200 eyes, are shown in Watson (2015, p. 15), based on Thibos, Hong, Bradley, and Cheng (2002).

**Figure 1.**
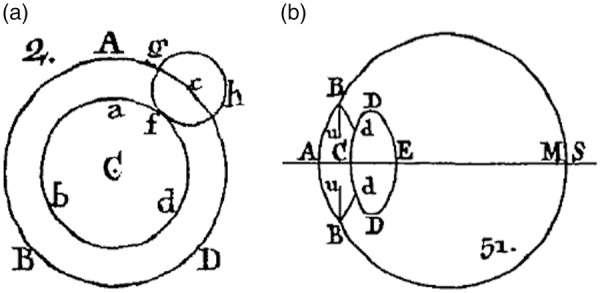
(a) Example of a circle of dissipation from insufficient accommodation in Jurin (1738, Figure 2). The circle, with center *c*, originates at the border of a ring stimulus with center *C*. (b) Jurin was aware of the eye’s basic anatomy, as shown by his sketch of the eye (Jurin, 1738, Figure 51).

**Figure 2.**
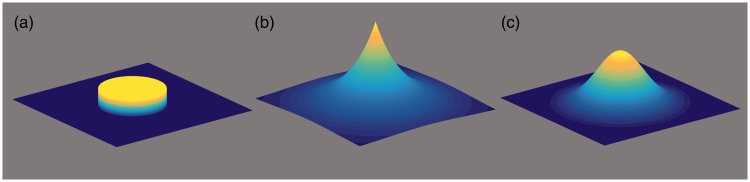
Various point-spread functions (PSFs) that lead to blur. (a) Slightly simplified PSF for defocus resulting from inaccurate accommodation or myopia. Note that the actual PSF on the retina is a superposition of that with influences from other factors. (b) PSF for a 2D low-pass filter in analogy to a first-order time-domain low pass. (c) A Gaussian PSF.

The role of the blur disk for blur, myopia, and accommodation has long been known. Jurin (1738) uses it in his *Essay on distinct and indistinct vision* when he describes ways to degrade the retinal image, from *perfectly distinct* (meaning perfectly in focus) to *imperfectly distinct*, meaning somewhat out of focus but perceptually still appearing as distinct. One of the methods used by Jurin was simply to bring the stimulus closer to the eye than accommodation would allow, such that the retinal image is out of focus.^1^ Figure 1 is an example from Jurin’s essay where the observer looks at a ring stimulus, fixating a point (c) on the ring’s outer circumference. The *pencil of rays* emanating from that point will be *dissipated* into a *circle of dissipation* (Jurin’s term for the blur disk), drawn, in the figure, into the stimulus (Jurin, 1738; Figure 2; here Figure 1).^2^ Jurin performs calculations with the *radius of dissipation*; he argues, for example, that the penumbra in a certain example has twice the thickness of the radius, and he describes how to measure the radius on that basis perceptually.^3,4^ He does not actually derive the size of the radius, however, even though he is aware of the role of the pupil size and lens curvature. Jurin had a good understanding of the eye’s anatomy and optics (Figure 1(b); cf. Strasburger & Wade, 2015; Wade, 2004).

To set dioptric blur apart from other kinds of blur, Figure 2 shows several types of a PSF^5^ that underlie various kinds of blur. The cylindrical PSF in Figure 2(a) is a simplified version of what happens optically; the exponential in Figure 2(b) is an illustration of a two-dimensional (2D) low-pass analog to the simplest low-pass filter in the time domain (it has no natural counterpart in 2D); and the Gaussian in Figure 2(c) is typically used for modeling low-pass filtering in the neural pathway, or, respectively, a difference of Gaussians for bandpass filtering (Daugman, 1980; Malik & Perona, 1990; Rose, 1979).

## Spurious Resolution

**Figure 3.**
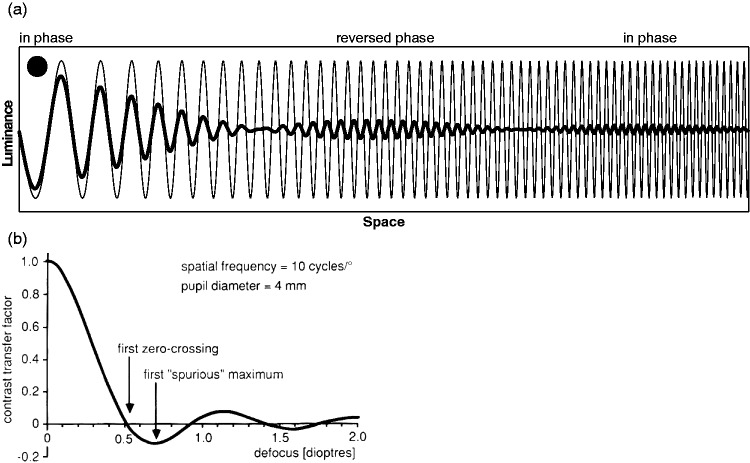
Illustration of spurious resolution. Top: The effect of defocus on a frequency-sweep sine-wave grating. The original grating (thin line) was convolved with a constant kernel of fixed diameter (represented by the disk in the upper left). The resulting defocused grating exhibits zero crossings of the envelope that separate segments that are in-phase with the original grating from segments with reversed phase. Bottom: The contrast transfer factor (i.e., real part of the optical transfer function) for a defocused periodic sinusoidal grating. At about 0.5 *D* of defocus the image contrast is zero. With higher spatial frequency, however, the grating reappears with inverted phase and lower contrast. From Bach et al. (1989; Figure 2). Note that the contrast transfer factor as used here is not directly related to the contrast transfer function, which is commonly defined for square wave gratings (Boreman, 2001).

The importance of choosing the proper type of blur becomes obvious when considering spurious resolution. This is a phenomenon long known in optical instrumentation (Hotchkiss, Washer, & Rosberry, 1951; Smith, 1982b); it may be illustrated by applying dioptric blur to a frequency sweep pattern, like the one in Figure 3(a), where spatial frequency increases from left to right. For a given defocus, grating contrast first decreases with increasing spatial frequency (as expected) reaching zero contrast at a certain spatial frequency (Figure 3(b)). With spatial frequency increasing further, however, the grating becomes visible again albeit with inverted phase. This is followed by another zero-crossing of contrast, followed by another spatial frequency interval with nonzero contrast and the original phase preserved. Such alternation of reversed and preserved phase continues up to the cut-off frequency of the optical system. Consequently, high spatial frequencies are detectable under dioptric blur, both psychophysically and when used as a stimulus for objective acuity testing (Bach, Waltenspiel, & Schildwächter, 1989; Heinrich, Lüth, & Bach, 2015; Smith, 1982b). This is the case even when the PSF is much wider than the period of the grating. High-acuity results, obtained with periodic patterns like sine-wave gratings, must thus not be taken at face value. Gaussian blur, in contrast, cannot evoke spurious resolution because the Fourier transform of a Gaussian remains a Gaussian and is always positive.

**Figure 4.**
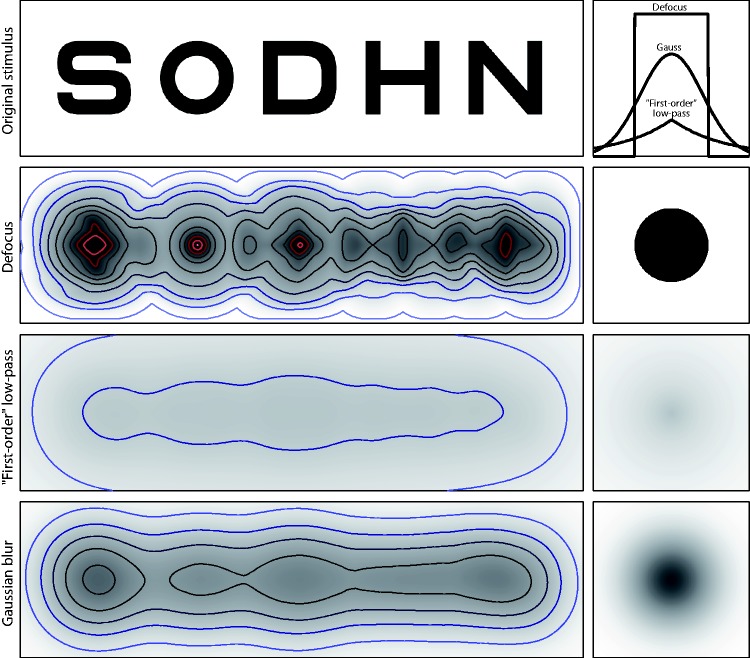
Effect of blur on Sloan letters. Top row: original, unblurred letters, together with point-spread-function profiles (right) for the lower rows. FWHMs of the three PSFs are equal. Note that PSF amplitudes are necessarily different since their volume needs to be normalized to unity (light is neither added nor lost). Second row: letters with dioptric blur simulated by using a disk with a diameter equal to the letter height as blur kernel. The effect of spurious resolution is so strong that the blurred letters look quite unlike their original. Third row: PSF with exponential drop-off (analogous to a first-order low-pass filter). Energy is spread over a wide spatial range, such that amplitude is rather low. Bottom row: letters with simulated Gaussian blur. For display, blurred images were increased in contrast to enhance the visibility of structures. Isolumes for all three patterns represent luminance steps of 7 percentage points (white = 100%). The gray scale representation of the PSF in the right column uses a different scale than the blurred images.

Dioptric blur is not unique in its ability to induce spurious resolution (Harding, 1977; Koenderink, 1984); Gaussian blur, however, is unique in avoiding spurious resolution (Koenderink, 1984). It is also obvious that the effect is not limited to gratings as in Figure 3(a), and related phenomena even occur with nonperiodic stimulus types, such as optotypes (Figure 4; see also Thorn & Schwartz, 1990; Wolf & Angerstein, 1978). The likely difference, however, between the effect on gratings (measuring resolution acuity) and the effect on optotypes (measuring recognition acuity; Heinrich & Bach, 2013; Strasburger & Rentschler, 1996) appears to be that, while the orientation of the grating can still be judged under the regimen of spurious resolution, the observer cannot make sense of the patchy image of the optotype that results from the phase shifts associated with spurious resolution (Bach et al., 1989; cf. Yellott & Yellott, 2007). However, there may be enough information left in the dioptrically blurred letter stimuli to allow for differentiation between letters after sufficient practice (Heinrich, Krüger, & Bach, 2011). The role of spurious resolution in reading is not yet clear (Chung, Jarvis, & Cheung, 2007). To avoid spurious resolution, artificial pupils with Gaussian aperture have been proposed (Fry, 1953).

The above points illustrate that the choice of the proper blur kernel is of practical relevance when dioptric blur-related vision impairments are considered. The considerable qualitative difference between blur obtained with different blur kernels, as illustrated in Figure 4, also demonstrates the limitations of those approaches that attempt to combine multiple sources of blur into one equivalent Gaussian blur kernel (e.g., Coppens & van den Berg, 2004).

More generally, different visual impairments are associated with different kinds of image degradation. Myopia is closely related to dioptric blur. Cataract, on the other hand, involves wide-angle scattering (de Wit, Franssen, Coppens, & van den Berg, 2006; de Waard, IJspeert, van den Berg, & de Jong, 1992; Elliott, Bullimore, Patla, & Whitaker, 1996). Amblyopic vision, although appearing blurred to the subject, is not validly simulated by blur kernels altogether, as it appears to be associated with perceptual distortions and image fragmentation (Piano, Bex, & Simmers, 2015; Sireteanu, Bäumer, Sârbu, & Iftime, 2007; Sireteanu, Lagrèze, & Constantinescu, 1993). This is the likely reason why there is a dissociation in amblyopia between optotype acuity and grating acuity (or pattern acuity), both as measured psychophysically (Bach, Strahl, Waltenspiel, & Kommerell, 1990; Friendly, Jaafar, & Morillo, 1990; Gwiazda, 1992; Kushner, Lucchese, & Morton, 1995) or in *objective* acuity tests based on pattern VEPs (Heinrich, Bock, & Bach, 2016; Wenner, Heinrich, Beisse, Fuchs, & Bach, 2014).

Returning to dioptric blur, is a geometrical approximation of the dioptric PSF sufficient or is a wave-optics approach required? As Yellott and Yellott (2007) illustrate, the answer to that depends, to a large degree, on the relative effects of defocus and diffraction, with the latter being related to pupil size. Various higher order aberrations (see, for instance, Schaeffel, 2006) and the Stiles-Crawford effect (Stiles & Crawford, 1933) may also come into play. However, as Artal (2014) points out, in eyes with normal optics, the amount of higher order aberrations and visual acuity are not related; it may even be that the normal pattern of aberrations provides the best performance (Artal et al., 2004). Defocus is further the main source of degradation in the retinal images in most persons; the relative impact of aberrations on image quality is comparable only for amounts of defocus below 0.25 *D* (Artal, 2014, pp. 351–352). Thus higher order aberrations are not pursued here.

**Figure 5.**
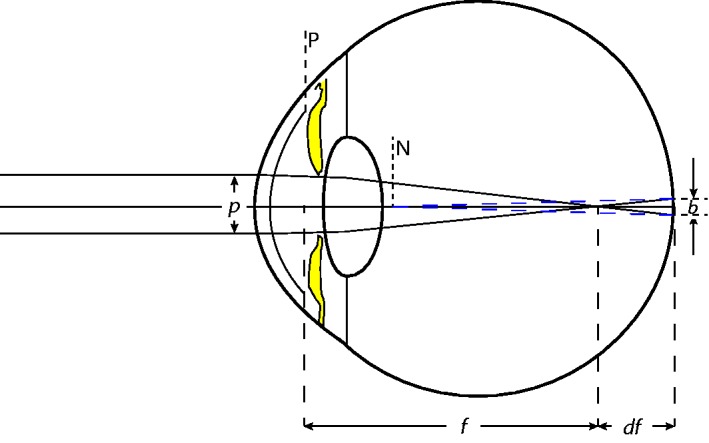
Simplified ray-path calculation of the blur-disk diameter from pupil diameter, assuming thin lenses and disregarding aberration and diffraction. *p* = Effective pupil diameter; *f* = focal length of the optical system (cornea and lens) within the eye; *df* = focal length error; *b* = blur-disk diameter; *P* = principal point; *N* = nodal point.

Equations that relate the blur-disk diameter to defocus like the one given below have been derived or cited earlier (Smith, 1982a, Equations (10) and (15); Dehnert, Bach, & Heinrich, 2011; Jacobs, Smith, & Chan, 1989; Ravikumar, Sarver, & Applegate, 2012; Smith, 1991). In Figure 5, we present an intuitive approach, slightly different to that in, for example, Smith, 1982a^6^ or Atchison and Smith (2000).

## Blur-Disk Diameter and Pupil Diameter

Unlike the case for the Gaussian, the dioptric blur-disk’s diameter is reasonably well defined since the luminance-times-blur-disk volume is (approximately) cylindrical (Figure 2(a)). Under a few simplifying assumptions its diameter depends—in a surprisingly straightforward way—on defocus and pupil diameter: The blur-disk diameter *b°,* in degrees of visual angle, is (as will be derived later) given by

(1) $$b^{\circ }=\frac{180}{\pi }10^{-3}p_{\mathrm{mm}}D,\text{or},\text{simply}b^{\circ }=0.057p_{\mathrm{mm}}D,$$

where *p_mm_* is the pupil diameter in mm, and *D* is defocus in diopters (D). The equation allows straightforward calculation of the blur kernel. This is well known (Atchison & Smith, 2000, pp. 82–84; Smith, 1982a), but here is a simplified derivation for convenience:

Figure 5 shows the enveloping rays from a far-away point source in a myopic eye, that is, an eye in which the focus lies before the retina (by a distance *df* ). Disregarding the differences in the optic media within the eye and with a few further definitions—

Focal length in the vitreous body (m): *f* (approximately 22.3 mm = 2.23 × 10^−2^ m)

Accommodation error of focal length (m): *df*

Accommodation error (Diopters = m^−1^): *D*

Distance of the retina (m): *f + df*

Effective pupil diameter (m): *p*

Retinal blur-disk diameter (m): *b*

Refractive index: *n* (approximately 1.336; Le Grand, 1968, p. 49)

—we have, by the definition of refractive power (in Diopters),

(2) $$D=\frac{n}{f+\mathrm{df}}-\frac{n}{f},$$

which, on a common denominator, is

(2a) $$D=-\frac{n\mathrm{df}}{f(f+\mathrm{df})}.$$

By the Intercept Theorem we have

(3) -b/df=p/f,

which, when solved for the blur-disk diameter *b*, results in

(4) $$b=-p\frac{\mathrm{df}}{f}.$$

For converting the linear blur-disk diameter *b* on the retina from meters to visual angle in radians, note that the angular size is to be taken from the eye’s back nodal point (*N* in the figure and blue dashed lines), whose distance from the retina is *d_N_* = 16.68 mm (Le Grand, 1968, p. 49). The value of *(f+df)*, on the other hand, is measured from the back principal point (*P*) which is at a distance of *d_P_* = 22.29 mm from the retina. The ratio of these two values is the refractive index, *n* = 1.336 (also Le Grand, 1968, p. 49):

(5) $$d_{P}/d_{N}=n$$

Since we assume paraxial approximations (i.e., small angles and thus tan *ϕ* = *ϕ*) we have, by definition,

(6) $$b_{\mathrm{rad}}=\frac{b}{d_{N}}=\frac{b}{d_{P}}n=\frac{b}{f+\mathrm{df}}\;n$$

Inserting Equation (4),

(7) $$b_{\mathrm{rad}}=-p\frac{\mathrm{df}}{f}\frac{n}{f+\mathrm{df}}$$

and Equation (2a), we arrive at

(8) $$b_{\mathrm{rad}}=\mathrm{pD}.$$

This is almost the desired equation except that the pupil diameter refers to a size within the eye which is not readily available. The size *p* of the pupil as seen from the outside, called the *entrance pupil*, is slightly larger than the actual size, since the physical pupil is seen through the cornea, that is, through a magnifying lens. However, since the lens is close to the nodal point, the enlargement is small and we will neglect it here. Thus, the size of the blur disk on the retina, in radians, is given to a good approximation by the above equation (Equation 8), where *p* is the size of the entrance pupil, that is, the size as seen from the outside, and *D* is defocus in the air. It is a linear relationship between angular blur-disk diameter and defocus, with the slope given by the pupil diameter.

Often, we are in a situation where we do not know the accommodation error in diopters but do know by how much an image is too near or too far from the eye. Following James Jurin’s example, find your personal near point and then, gradually, move the object closer. Or, if you are myopic, take off your glasses and move the object a little farther away than your far point. The image (from the well-focused case) will then, effectively, be convolved by a blur disk, the linear size *b_stim_* of which is given by

(9) $$b_{\mathrm{stim}}=p|\frac{d_{\mathrm{foc}}-d_{\mathrm{stim}}}{d_{\mathrm{stim}}}|,$$

where *p*, as before, is effective pupil diameter, *d_foc_* is the distance of the focal plane (e.g., the near point), and *d_stim_* is the distance of the stimulus. Like Equation (3), this follows directly from the Intercept Theorem (similar triangles), now applied, however, to the exterior space instead of to the interior of the eye (cf. Smith, 1982b, p. 15; Equation 16).

## Examples

**Figure 6.**
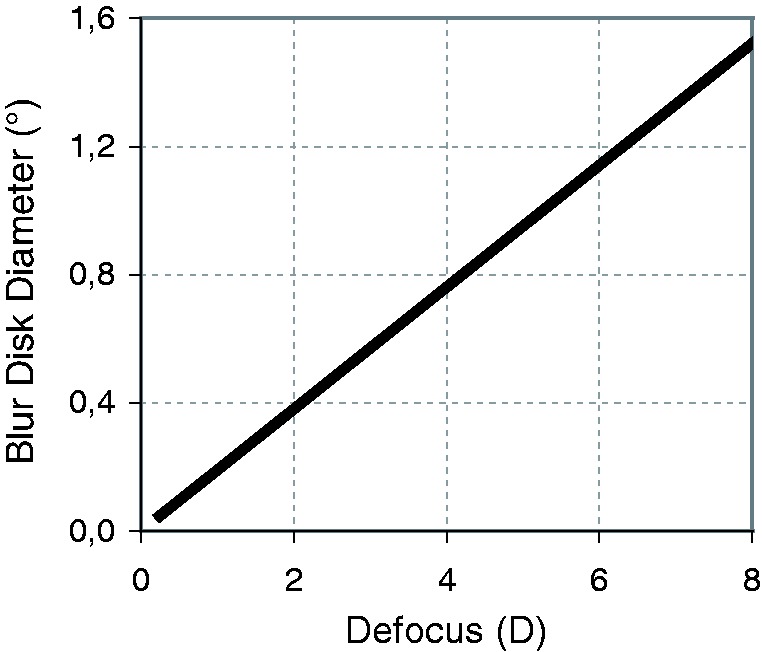
Blur-disk diameter versus defocus; an example for Equation (1), with pupil size set at 3.34 mm. That pupil size is the one expected for a 40-year-old subject watching a field of 28° diameter at 100 cd/m^2^ luminance (following Watson & Yellot, 2012).

Figure 6 shows the linear relationship of Equation (1) (or, with rescaling, of Equation (8)) for a quick look at what blur-disk diameter to expect at a certain defocus for an adult subject. Workplace progressive addition lenses, for example, with half a diopter undercorrection for far vision, would thus give a blur disk of approximately 0.1° = 6′ visual angle, disregarding other factors. Note that with less than about ¼ D defocus, factors other than defocus become more prominent in determining blur (Artal, 2014).

**Figure 7.**
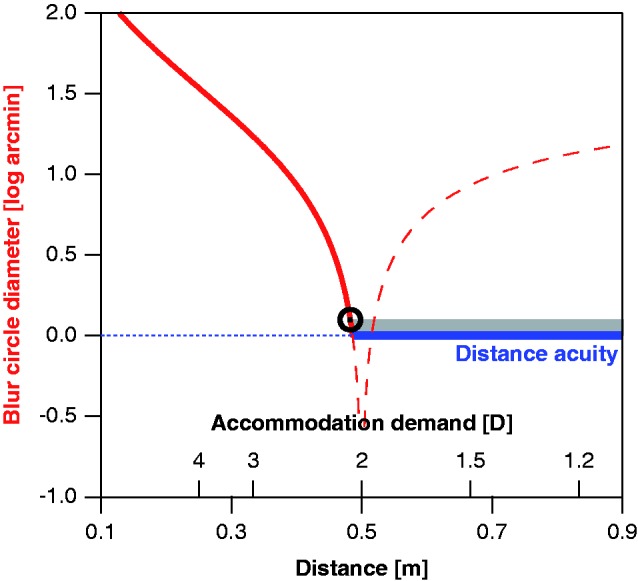
The subjective near point of accommodation (black circle) for an observer with natural lens, derived from the blur-circle (PSF) diameter (left ordinate) in a simplified model. Moving along the solid blue trajectory in the graph from right to left corresponds to an approaching target. With proper accommodation, no dioptric blur occurs (blue line); acuity in this condition would be referred to as the distance acuity. Proximal to the limit of accommodation (here assumed to be at 2D as in a typical 50-year-old emmetropic observer), dioptric blur increases and degrades acuity (solid red line). The dashed red line represents the blur-circle diameter in the case of a fixed focal length, as in a person with a monofocal intraocular lens, assumed to be in-focus at 50 cm.

As a further example, let us consider the near point for an observer with a natural lens—the closest distance for an object to appear in focus with a given accommodation capability (Figure 7). With proper accommodation, no dioptric blur occurs; this results in constant acuity as long as distance is large enough to be within the range of accommodation. That acuity along the blue segment is referred to as the *distance acuity*, here set to a value equivalent to the acuity achieved with a blur circle of 1 arcmin diameter. Proximal to the limit of accommodation, dioptric blur increases and degrades acuity (solid red line). When the near point is determined by deciding when the approaching target starts appearing blurred, the just-noticeable difference in blur (indicated by gray shading above the blue line) results in a small additional shift in the subjective near point. The dashed red line represents the blur-circle diameter in the case of a fixed focal length, for example in a person with a monofocal intraocular lens, assumed to be in-focus at 50 cm. This also approximates the situation when wearing single-vision reading glasses in advanced presbyopia with little or no residual accommodation. Computations were performed for a pupil size of 5 mm. In reality, the transition between distance acuity and blur-affected near acuity is less abrupt than depicted, as the switch between the two operating ranges is not sudden.

## Visual Acuity and Defocus

**Figure 8.**
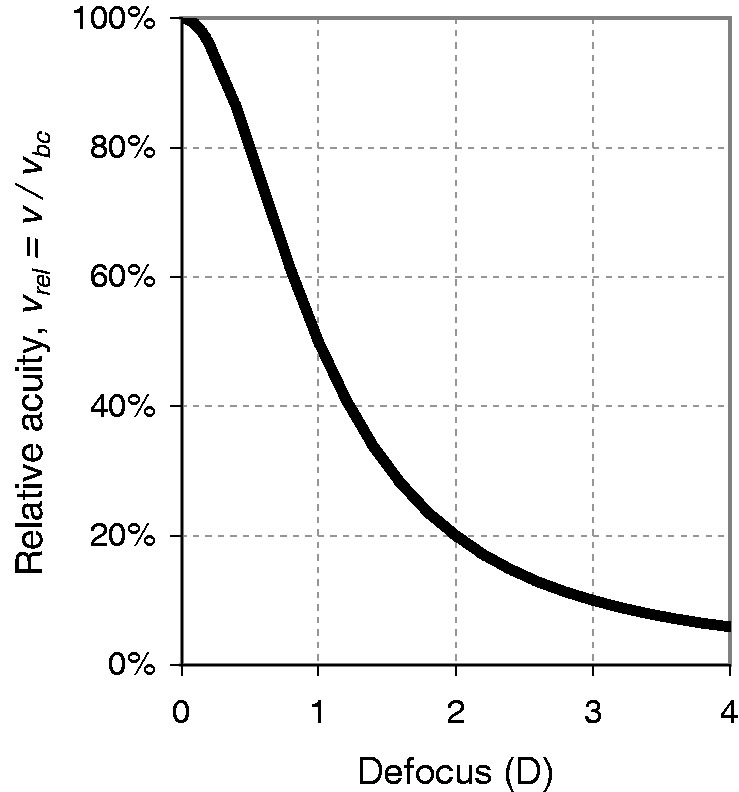
Effect of defocus on relative acuity, according to Blendowske’s (2015) empirical model, *V_rel_* = *V/V_bc_* = 1/(1*+D^2^*).

Let us finally turn to a question that will quickly arise in the context of blur: How is acuity affected by defocus? Acuity will obviously be best when blur is minimal (see Artal, 2014, for a qualification of that assertion), so a better question is how much is acuity *reduced* by defocus? Let *v_bc_* denote the visual decimal acuity achieved with the best correction in place, and *v* the acuity with blur present. We are then seeking how the degradation factor *v/v_bc_* depends on defocus, that is, on the spherical error, which we have called *D* above. Here, Blendowske (2015) has derived a surprisingly simple empirical relationship for the reduction of acuity:

(10) $$v/v_{\mathrm{bc}}=\frac{1}{1+D^{2}},$$

where *v*/*v_bc_* is visual acuity (decimal or Snellen fraction) relative to the best-corrected case, and *D* is the spherical error in diopters (Figure 8). Blendowske’s equation (which in his publication also includes cylindrical refractive errors which we omit here) was inspired by Raasch (1995), who had fit a second-order polynomial to a large set of empirical data with natural pupil sizes, relating acuity to (spherical and cylindrical) refractive error. Blendowske extended that data set to include even more published data, in particular data for small refractive errors, again with natural pupils of diameters in the range from 2 to 5 mm. It turned out that, by estimating *relative* rather than absolute acuity, Blendowske obtained his much simpler Equation (10), which (unlike Raasch’s equation or the Equations (1) or (8) above) also works well for low refractive errors, down to zero diopters. Note that the equation is based on empirical data, not on physical modeling, and thus naturally includes the influences of higher order aberrations and diffraction. Note further that pupil size, although it influences acuity, does not appear in the equation. Nevertheless, the fit to the data is very good, also at small values of *D*, with a regression standard error of 0.046 log units; residual error mostly stemmed from not controlling for pupil size. As a practical example, mis-accommodation by ½, 1, 2, or 3 *D* in a subject having 1.0 decimal acuity will degrade that to a value of 0.8, 0.5, 0.2, or 0.1, respectively.

For small values of defocus the equation simplifies further, to

(11) $$v/v_{\mathrm{bc}}\approx 1-D^{2}\text{forsmall}D(6\%\text{errorbelow}1/2).$$

Since the graphs for the (empirical) Equations (10) and (11) have a ½horizontal tangent at zero diopters, there is rather little change of acuity with blur for small blur values (<¼ D), a familiar finding in everyday practice.

Visual acuity (as measured with grating stimuli) is essentially determined by the high-frequency end of the contrast sensitivity function (effects of blur also extend to other parts of the contrast sensitivity function of course). This is quantified by the corresponding modulation transfer function (see, for instance, Smith, 2000, Figure 11.16 on p. 378).

## Conclusion

We have illustrated and discussed choosing the proper blur kernel to simulate visual degradation. In the case of defocus, this is a simple disk. Different blur kernels may produce qualitatively different images. In particular, Gaussian blur does not introduce spurious resolution and related effects and is thus fundamentally different from the blur that is associated with typical optical problems. The blur-disk’s diameter, except for small values of defocus, is proportional to defocus in diopters and pupil size; its linear size in a stimulus is proportional to the stimulus’ relative distance from the near or far point. Degradation of visual acuity from its best-corrected value is related to defocus blur by a simple (inverse) second-order equation in a wide range of defocus including small values.

## Notes

1 “…bring the book by degrees so near, as that the letters of the smallest print now begin to appear a little confused,… Here, it is manifest from the less distinct appearance of the smaller print, that at this distance the rays of each pencil [of rays] are not accurately united in a sensible [i.e., sensitive] point of the *Retina*…” (Jurin, 1738, p. 116)

2 “17.… the rays of each pencil issuing from the object cannot be united but at a point beyond the *Retina;* consequently, the rays of each pencil will occupy a circular space upon the Retina.… [L]et the circle… represent that circular space upon the Retina, which is taken up by one of the extreme pencils of rays issuing from the object. This circle fghc we shall call the *circle of dissipation,* because the rays of a pencil, instead of being collected into the central point c, are dissipated all over this circle: And the radius of this circle… we shall for the same reason call the *radius of dissipation*.” (Jurin, 1738, p. 117)

3 “Upon white paper draw the circumference of a circle with a strong black line and place the paper by guess about the farthest distance at which your eye can see an object distinctly [Jurin assumes a myopic person here]. Then retiring gradually farther from the paper, observe at what distance the white circle… appears equal in breath to the *penumbra* on either side of it. At that distance the *radius of dissipation* is nearly equal to half the radius of the *true image* of the circle.” (Jurin, 1738, p. 131)

4 Jurin (incorrectly) believed that accommodation works symmetrically, accommodating distances both nearer to and farther from what he calls a *natural distance* of 33 in.: 137.… Whence it is reasonable to conclude, that the natural distance is such, as that no greater change of conformation is required to reduce it to the least distance, than to increase it to the greatest distance at which we can see distinctly. (Jurin, 1738, p. 141)

5 A point-spread function is the 2D equivalent of an impulse-response function. It allows an easy derivation of the resulting image, that is, here the blurred image: According to the Convolution Theorem, the blurred image is simply the convolution of the point-spread function with the original image.

6 A few misprints in Smith (1982a) hinder the understanding of Derivation no. 1 presented there (the derivation inside the eye, corresponding to the one here): (1) It should be δl′ instead of φl′ on the right side in Equation (4), so that Equation (3) can be inserted in it. (2) The sign of the accommodation level L_0_ in Equation (6a) is incorrect and the equation should be (F_e_)_R_ = F_e_ + L_0_ instead. The term then cancels with the denominator in Equation (5). (3) The power of the relaxed eye (F_e_)_R_ is said (a few lines further down) “to be equivalent to” the distance of the back nodal point from the retina, N′F′. This is shorthand for saying that one is the inverse of the other, that is, (F_e_)_R_ = 1/N′F′. Typically, however, the power of the relaxed eye (F_e_)_R_ is considered as taken from the back principal point H′, not the back nodal point N′: (F_e_)_R_ = n′/H′F′. Yet the ratio of the two distances equals the refractive index, that is, H′F′/N′F′ = n′, so the two assertions are equivalent.
